# Supplementary material for: A scoping review of portable sensing for out-of-lab anterior cruciate ligament injury prevention and rehabilitation
Source: NPJ Digit Med. 2023 Mar 18;6:46. doi: 10.1038/s41746-023-00782-2 (PMC10024704; doi:10.1038/s41746-023-00782-2)
Supplement: Supplementary file 1 — Supplementary Table 1 [file 41746_2023_782_MOESM1_ESM.pdf]

Supplementary Table 1 The Appraisal tool for Cross-Sectional Studies (AXIS).

No.	Question	Category
Q1	Were the aims/objectives of the study clear?	Quality of reporting
Q2	Was the study design appropriate for the stated aim(s)?	Quality of study design
Q3	Was the sample size justified?	Quality of study design
Q4	Was the target/reference population clearly defined? (Is it clear who the research was about?)	Quality of reporting
Q5	Was the sample frame taken from an appropriate population base so that it closely represented the target/reference population under investigation?	Quality of study design
Q6	Was the selection process likely to select subjects/participants that were representative of the target/reference population under investigation?	Potential biases
Q7	Were measures undertaken to address and categorise non-responders?	Potential biases
Q8	Were the risk factor and outcome variables measured appropriate to the aims of the study?	Quality of study design
Q9	Were the risk factor and outcome variables measured correctly using instruments/ measurements that had been trialled, piloted or published previously?	Potential biases
Q10	Is it clear what was used to determined statistical significance and/or precision estimates? (eg, p values, CIs)	Quality of reporting
Q11	Were the methods (including statistical methods) sufficiently described to enable them to be repeated?	Quality of reporting
Q12	Were the basic data adequately described?	Quality of reporting
Q13	Does the response rate raise concerns about non-response bias?	Potential biases
Q14	If appropriate, was information about non-responders described?	Potential biases
Q15	Were the results internally consistent?	Potential biases
Q16	Were the results for the analyses described in the methods, presented?	Quality of reporting
Q17	Were the authors' discussions and conclusions justified by the results?	Quality of study design
Q18	Were the limitations of the study discussed?	Quality of reporting
Q19	Were there any funding sources or conflicts of interest that may affect the authors’ interpretation of the results?	Quality of study design
Q20	Was ethical approval or consent of participants attained?	Quality of study design
